# Multiple Consequences of a Single Amino Acid Pathogenic RTK Mutation: The A391E Mutation in FGFR3

**DOI:** 10.1371/journal.pone.0056521

**Published:** 2013-02-20

**Authors:** Fenghao Chen, Sarvenaz Sarabipour, Kalina Hristova

**Affiliations:** Department of Materials Science and Engineering, Johns Hopkins University, Baltimore, Maryland, United States of America; University of Rome, Italy

## Abstract

The A391E mutation in fibroblast growth factor receptor 3 (FGFR3) is the genetic cause for Crouzon syndrome with Acanthosis Nigricans. Here we investigate the effect of this mutation on FGFR3 activation in HEK 293 T cells over a wide range of fibroblast growth factor 1 concentrations using a physical-chemical approach that deconvolutes the effects of the mutation on dimerization, ligand binding, and efficiency of phosphorylation. It is believed that the mutation increases FGFR3 dimerization, and our results verify this. However, our results also demonstrate that the increase in dimerization is not the sole effect of the mutation, as the mutation also facilitates the phosphorylation of critical tyrosines in the activation loop of FGFR3. The activation of mutant FGFR3 is substantially increased due to a combination of these two effects. The low expression of the mutant, however, attenuates its signaling and may explain the mild phenotype in Crouzon syndrome with Acanthosis Nigricans. The results presented here provide new knowledge about the physical basis behind growth disorders and highlight the fact that a single RTK mutation may affect multiple steps in RTK activation.

## Introduction

Crouzon syndrome with Acanthosis Nigricans is an autosomal dominant growth disorder which affects 1 out of every 25, 000 births worldwide [1], [2]. Crouzon syndrome is a craniosynostosis, characterized by premature fusion of the skull and facial bones, which prevents normal skull growth in infants [3]. The phenotypic features include wide-set bulging eyes and underdeveloped upper jaw, and in some cases, hearing loss. The craniosynostosis phenotype occurs together with a skin disorder, Acanthosis Nigricans, characterized by dark, thick, velvety skin in body folds and creases.

Crouzon syndrome with Acanthosis Nigricans has been linked to the A391E mutation in the transmembrane (TM) domain of Fibroblast growth factor receptor 3 (FGFR3) [1], [4]. FGFR3 belongs to the receptor tyrosine kinase (RTK) family and transduces biochemical signals by lateral dimerization in the plasma membrane, followed by receptor phosphorylation and stimulation of downstream signaling cascades [5], [6]. FGFR3 signaling is critically important for normal cellular growth, proliferation, and differentiation [5], [7]–[9]. In the skeletal system, FGFR3 exerts negative regulation over bone growth, and FGFR3 over-activation interferes with normal growth and development [10], [11].

Previous biophysical studies have shown that the A391E mutation stabilizes the isolated FGFR3 TM domain homodimers in lipid bilayers by −1.3 kcal/mole [12]. Based on molecular modeling, the stability of the mutant dimers has been proposed to increase due to Glu391-mediated hydrogen bonding [12]. The A391E mutation further enhances the activation of full-length FGFR3 in HEK 293T cells in the absence of ligand by −1.7 kcal/mole [13]. Thus, the effect of the mutation on FGFR3 activation in the absence of ligand is now established. A question remains, however, if the A391E mutation affects the response of FGFR3 to ligands. FGFR3 binds to ligands from the *fgf* family, with the aid of heparin or heparan sulfate proteoglycan (HPSG) [5], [14], [15]. Ligand binding is believed to stabilize FGFR3 dimers and possibly alter their structure and activity [16].

Here we compare the responses of wild-type FGFR3 and the A391E mutant to the ligand *fgf1* over a wide range of *fgf1* concentrations. We analyze FGFR3 activation using Western blots and a simple physical-chemical model describing FGFR3 activation as a process involving dimerization, ligand binding, and phosphorylation [17]. We confirm that the mutation enhanced FGFR3 dimerization, as previously proposed [12], [13]. We also demonstrate that the mutation increases the phosphorylation efficiency within both unliganded and ligand-bound dimers, without affecting the strength of ligand binding. The results show that a single mutation can affect different events controlling RTK activation, highlighting the complexity of RTK signaling in health and disease.

## Materials and Methods

### Plasmids

The plasmid encoding human wild-type FGFR3 (FGFR3/WT) in the pcDNA 3.1+ vector was a generous gift from Dr. D.J Donoghue, UCSD. The mutant FGFR3 plasmid (FGFR3/A391E) was produced using Rapid Change Mutagenesis Kit XL II (Stratagene).

### Cell Culture and Transfection

Human Embryonic Kidney 293 T (HEK 293 T) cells were obtained from the laboratory of Prof. M. Edidin (JHU), and were cultured at 37°C with 5% CO_2_ for 24 hours. The cells were transfected with plasmids encoding FGFR3/WT and FGFR3/A391E using Fugene 6 (Roche), following the manufacturer protocol.

### Western Blots

HEK 293 T cells were cultured for 24 hours following transfection, starved in serum-free medium for 24 hours and then treated with lysis buffer (25 mM Tris-Cl, 0.5% TritonX-100, 20 mM NaCl, 2 mM EDTA, 2 mM Na_3_VO_4_ and protease inhibitor, Roche Applied Science). The lysates were collected following centrifugation at 15,000 g for 15 minutes at 4^o^C and loaded onto 3–8% NuPAGE® Novex® Tris-Acetate mini gels (Invitrogen, CA). Because of the large number of samples that had to be compared, the lysates in each independent experiment were loaded onto two gels, and the proteins in the two gels were transferred simultaneously onto a nitrocellulose membrane, and blocked using 5% milk in TBS. FGFR3 total protein and phosphorylation levels were probed with antibodies against FGFR3 (H-100; sc-9007, Santa Cruz Biotechnology) and phosphorylated FGFR (anti-Y653/654; Cell Signaling Technology), respectively, followed by anti-rabbit HRP conjugated antibodies (W4011, Promega). The membranes were incubated with substrate (Amersham ECL Plus™ Western Blotting Detection Reagent) for 2 minutes. The x-ray film was exposed for 3 minutes to image the anti-pFGFR antibodies, and for 1 minute to image the anti-FGFR3 antibodies.

### Titration with Ligand

Cells were starved for 24 hours in serum-free medium, before *fgf1* (Millipore, MA) was added at concentrations ranging from 5 ng/ml to 5000 ng/ml. After incubating for 10 minutes with ligand, the cells were lysed and analyzed by Western blots.

### Crosslinking

Dimeric receptors were cross-linked with the membrane impermeable crosslinker BS^3^ (Pierce). After starvation for twenty-four hour, cells were incubated with 2 mM cross-linker for 30 minutes to 1 hour at room temperature, and then quenched in 20 mM Tris-HCl for 15 minutes. After two rinses with ice-cold PBS, the cells were lysed and the receptors were detected using Western blotting. The cross-linked fraction was calculated as *S_D_/S = S_D_/(S_M_+S_D_)*, where *S_D_* is the intensity of the dimeric band and *S_M_* is the intensity of the monomeric band [18].

### Quantification of Western Blots

The Western blot films were scanned and quantified using ImageQuant TL. At least three independent experiments were performed in order to determine averages and standard deviations. For quantification, the amount of protein lysate loaded onto gels was adjusted such that all the band intensities were within the so-called linear range, with intensities proportional to the receptor concentrations (See Methods S1, Figure S1, and [18]).

### A Physical-chemical Model of FGFR3 Activation

It is well established that FGFR3 activity is regulated by FGFR3 lateral dimerization. Ligands, such as *fgf1*, influence the dimerization process by binding to the unliganded dimer and stabilizing the dimeric state. We have established a simple physical-chemical model, which provides an adequate description of FGFR3 activation [17]. In this model FGFR3 ligand-independent dimerization is followed by ligand binding, which converts unliganded into liganded dimers:
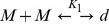
(1)

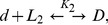
(2)In equations (1) and (2), *M* represents the monomer, while *d* and *D* represent the unliganded and ligand-bound FGFR3 dimers, respectively. The two reactions (1) and (2) are coupled: ligand binding depletes the unliganded dimers, and this in turn decreases the monomer concentrations. Unliganded and ligand-bound dimers are assumed to have different structures and activities. The ligand is assumed to be pre-dimerized prior to receptor binding [17], consistent with reports that *fgf1* dimerizes on heparan sulfates on the cell surface [15], [19].

The two reaction constants, *K_1_* and *K_2_*, governing ligand-independent dimerization and ligand binding, are defined as [17]:
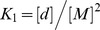
(3)


and
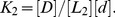
(4)


In addition, we write the mass balance equations for the total receptor concentration [*TR*] and the total ligand concentration [*TL*] as:
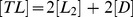
(5)

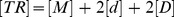
(6)Here we define the average receptor concentrations [*TR*], [*M*], [*D*], and [*d*] per cell. As the number of cells and the 3D ligand concentrations are known in the experiment, the average number of ligands per cell, [*TL*] is easily calculated. In the experiments the ligands can be found in three different states: free in solution, bound to heparin sulfate (HS) on the cell surface, and bound to the receptor dimer in the presence of HS. 2[*L_2_*] is the average number of ligands per cell that are not bound to the receptor dimers. The model does not consider explicitly the equilibrium between monomeric and dimeric ligands, as well as the equilibrium between ligands that are free in solution or bound to the cell surface [17].

Knowing the values of [*TL*] and [*TR*], and inputting values for the two reaction constants *K_1_* and *K_2_*, the four equations (3) through (6) yield the 4 unknowns [*L_2_*], [*M*], [*d*], and [*D*], as a function of total ligand concentration, [*TL*] for fixed *K_1_*, *K_2_*, and [*TR*]. The problem is reduced to a fourth-order equation for the monomer concentration [*M*], which yields a single real positive root, allowing us to calculate and predict the dimer concentrations, [*d*] and [*D*] (see [17] for details).

**Figure 1:**
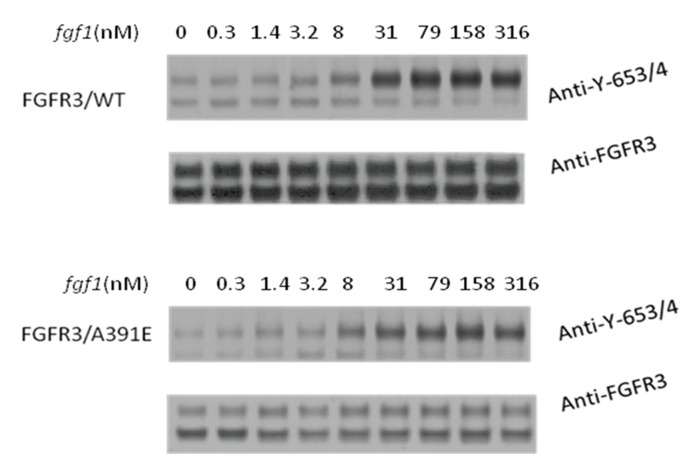
Western blots, showing the expression and activation of wild-type FGFR3 and the A391E mutant, as a function of ligand concentration. HEK 293 T cells were cultured for 24 hours following transfection, and starved in serum-free medium for 24 hours before stimulation with different concentrations of *fgf1*. Because of the large number of samples that had to be compared, the lysates in each independent experiment were loaded onto two gels, and the proteins in the two gels were transferred simultaneously onto a blotting membrane and incubated with antibodies as described previously [20]. FGFR3 expression was probed using anti-FGFR3 antibodies, and FGFR3 activation was probed using anti-Y653/4 antibodies. The latter antibodies are specific for two phoshorylated tyrosines in the activation loop of the FGFR3 kinase. Two bands are observed in each case, one corresponding to the 120 kDa immature FGFR3 located in the ER/Golgi, and a second one corresponding to the mature 130 kDa form located predominantly in the plasma membrane. Here we quantify and process only the 130 kDa bands.

In the dimer the kinase domains phosphorylate each other. We account for cross-phosphorylation using two additional parameters, the probabilities for phosphorylation in the unliganded and liganded dimers, Φ_d_ and Φ_D_. As discussed previously, these parameters describe how easy (or difficult) it is to phosphorylate specific tyrosines once the dimers are formed [13], [17], [20]. The concentration of phosphorylated receptors is then calculated according to:
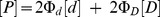
(7)The concentration of phosphorylated receptors [*P*] can be assayed using Western blots and antibodies that are specific for phosphorylated tyrosines. The measured phosphostaining, however, cannot yield real concentrations of phosphorylated receptors as it depends on the particular antibody used, on the exposure time, and on other experimental details. However, we have shown that the phosphorylated fraction can be measured if experiments are carried out over a very wide range of ligand concentrations, including very high ligand concentrations that drive all receptors into their liganded dimeric states as shown below in Figure 1. At saturating ligand concentrations, 2[*D*]*_sat_* = [TR] and therefore:
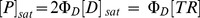
(8)Thus, the ratio of phosphostaining measured at particular ligand concentration to the phosphostaining at saturation yields the phosphorylated fraction of receptors:
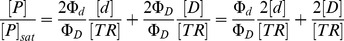
(9)The phosphorylated fraction is a parameter that is measurable, and here we measure it over a wide ligand concentration range. The experimental data can be fitted to the prediction given by equation (9), which depends on K1 and K2 (because [d] and [D] in equation (9) depend on K1 and K2, in accordance with equations (3) through (6)).

While the real values for the probabilities for phosphorylation, Φ_d_ and Φ_D_, cannot be determined, the ratio of the two probabilities can be calculated if the concentrations of phosphorylated receptors and dimeric receptors are measured. At saturating ligand concentrations, only liganded dimers are expected to be present and the concentration of phosphorylated receptors is given by equation (8). In the absence of ligand, the concentration of phosphorylated receptors is:
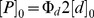
(10)Taking the ratio of (10) and (8), we obtain:
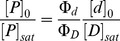
(11)Therefore:
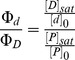
(12)The ratio [P]sat/[P]0 is measured using Western blotting as the ratio of anti-phospho staining intensities at saturating ligand concentration and in the absence of ligand. Dimeric fractions, and thus [D]sat/[d]0, however, are difficult to measure. Since direct methods to measure dimeric fractions in cellular environment are lacking, the dimeric fractions in the absence of ligand have been previously approximated with the ratio of cross-linked fractions at zero ligand and at saturating ligand concentrations [17], [20].

## Results

### Western Blot Analysis of Mutant FGFR3 Expression and Activationz

Previously we have studied the response of wild-type FGFR3 to the ligand *fgf1*, over a broad ligand concentration range (0–5000 ng/ml, 0–316 nM), in HEK 293 T cells [20]. Here we monitored the response of FGFR3 carrying the A391E mutation to *fgf1*, and we compared the ligand-induced activation of the mutant to that of the wild-type. In these experiments, HEK 293 T cells were transiently transfected with 1 µg of DNA encoding FGFR3/A391E. Cells were starved for 24 hours prior to ligand addition to remove the interference from the growth factors in the fetal bovine serum (FBS) and to induce accumulation of receptors on the plasma membrane [20]. Cells were then incubated with *fgf1* at concentrations ranging from 5 to 5000 ng/ml for 10 min before lysis and analysis by Western blots.

Unlike previous studies, here we carried out a direct side-by-side comparison of the activation of the wild-type and the mutant over a wide range of ligand concentrations. Because of the large number of samples that had to be compared, the lysates in each independent experiment were loaded onto two gels, and the proteins in the two gels were transferred simultaneously onto a blotting membrane and incubated with antibodies as described previously [20]. Typical Western blot results for the wild-type and the mutant are shown in Figure 1, when 1 µg of DNA was used for transfection. Two different antibodies were used to detect FGFR3 expression and activation in the Western blots. Total receptor expression was probed with anti-FGFR3 antibodies (H-100, sc-9007, Santa Cruz Biotechnology). FGFR3 activation was probed using anti-phospho-FGFR antibodies (anti-Y653/4, Cell Signaling Technology). Because of the different degrees of FGFR3 glycosylation, as expected, two bands were detected for both the wild-type and the mutant: the intermediate 120 kDa form that is sensitive to Endo-H and found in the ER/cis-Golgi, and the fully glycosylated mature 130 kDa form located predominantly in the plasma membrane [13], [21].

The intensities of the anti-FGFR3 bands in Figure 1 report on FGFR3 expression. We observe decreased anti-FGFR3 band intensities (and thus decreased expression) for the mutant, as compared to the wild-type, under identical transfection conditions. Since in this study we are interested in the fully glyscosylated 130 kDa form localized predominantly in the plasma membrane, we quantified the top band intensities as described in Materials and Methods. The ratio of the top band intensities, and thus the expression ratio of the 130 kDa mature form (mutant over wild-type), was determined as 0.31 for the experiment shown in Figure 1. From four independent experiments, the expression of the 130 kDa mutant was determined as 0.4±0.1 of the 130 kDa wild-type expression. This is consistent with previous FACS results that have demonstrated a difference in the plasma membrane concentrations of the wild-type and the mutant (but no measurable effect of the mutation on the fraction of transfected cells) under identical transfection conditions [13].

The intensities of the anti-Y653/4 bands in Figure 1 report on FGFR3 activation. The anti-Y653/4 antibodies are reactive to the two phoshorylated tyrosine residues, Y647 and Y648, in the activation loop of FGFR3 kinase domain. The phosphorylation of these critical residues is required for the activation of the kinase and the phosphorylation of other intracellular tyrosines [22], [23]. We see that only the mature FGFR3 located predominantly on the cell surface responds to *fgf1*, while the immature FGFR3 located in the ER is not affected. Comparing the activation of the wild-type and mutant 130 kDa isoforms in Figure 1 (see top anti-Y653/4 bands), we see that the staining intensities for the mutant and the wild-type are not dramatically different. Yet, the expression of the wild-type is much higher, such that the activation level (activity per receptor) for the mutant is higher. It therefore appears that the mutation increases the receptor activation over the entire ligand concentration range.

**Figure 2:**
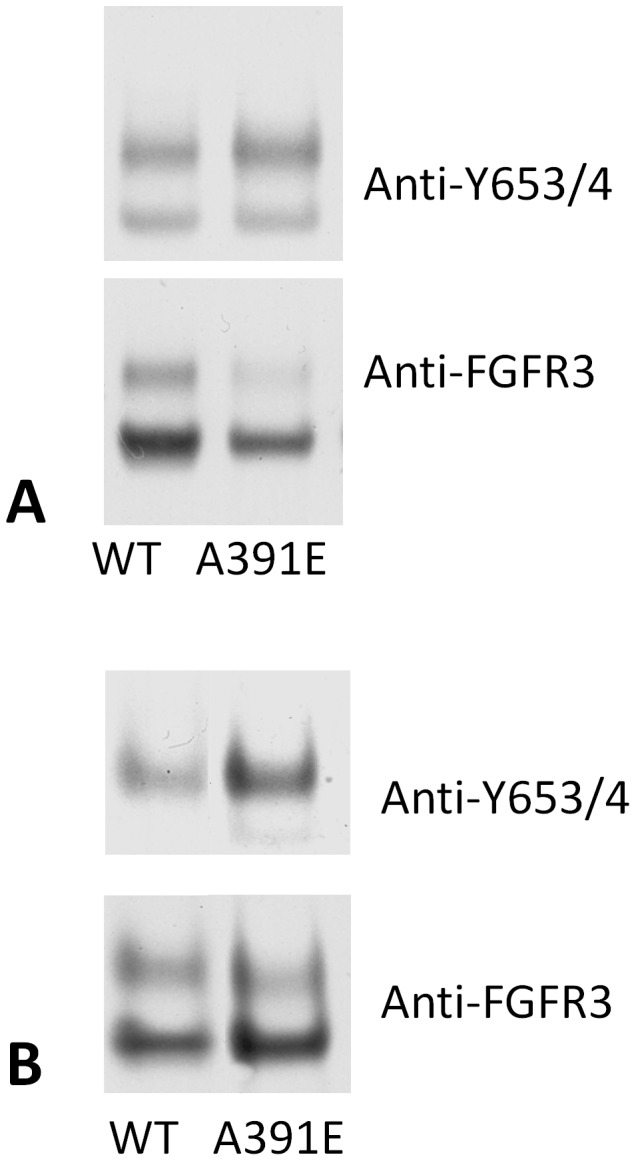
Side-by-side comparison of the expression and phosphorylation of the wild-type and the mutant. The phosphorylation of the mature 130 kDa form (top bands) is higher for the mutant, despite the fact that its expression is lower. (A) no ligand. (B) saturating ligand concentration.

As the expression of the wild-type and the mutant in the experiment shown in Figure 1 was quite different, we next doubled the amount of mutant DNA in order to decrease the expression difference, and compared the expression and phosphorylation of the receptors in the absence of ligand and in the presence of 316 nM *fgf1*. The results are shown in Figure 2, and they further demonstrate that the activation of the mutant is higher while its expression is lower, consistent with the conclusion from Figure 1.

### Effect of the A391E Mutation on FGFR3 Activation at High fgf1 Concentration

In Figure 1 we see that the activation of both wild-type and mutant FGFR3 increases as the ligand concentration is increased, until a plateau is reached for ligand concentrations higher than 1 µg/ml. This plateau is predicted for the model given by equations (1) through (9). Indeed, at very high ligand concentrations all receptors that are exposed to ligand and are capable of ligand binding should be in the liganded dimeric state. As discussed in Methods S1 and Figure S1, the Western blot protocols that we use ensure that the band intensities are always within the so-called linear range, i.e. the band intensities are proportional to the receptor concentrations [18], [21], [24]. Under these conditions the saturation we observe is not an experimental artifact, but a true saturation in receptor phosphorylation.

The fact that a plateau is reached in such experiments has allowed us previously to calculate active fractions at different receptor expressions [13]. As here we performed a side-by-side comparison of the wild-type and the mutant, the fact that plateaus are reached allows us to directly compare the phosphorylation states of the wild-type and mutant receptors when they are in the all-liganded dimeric state (see equation 8). Ultimately, this allows us to decouple the effect of the mutation on FGFR3 dimerization and on FGFR3 phosphorylation efficiencies in the dimers, as discussed below.

**Figure 3:**
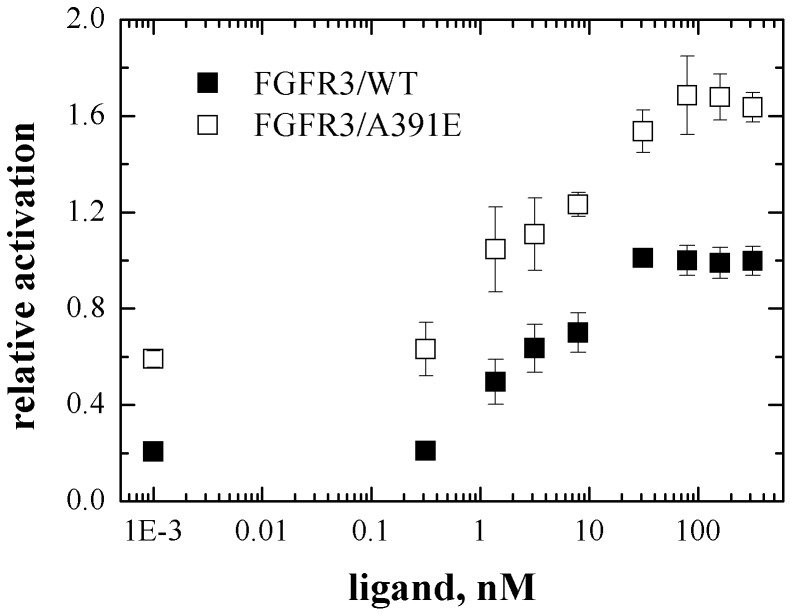
FGFR3 activation as a function of ligand concentration, on a relative but internally consistent scale. Data shown are averages and standard errors from 3 independent experiments (one of them is presented in Figure 1). HEK293 T cells were transfected with plasmids encoding FGFR3/WT or FGFR3/A391E. They were starved for 24 hours in serum-free medium, before *fgf1* (Millipore, MA) was added at concentrations ranging from 5 ng/ml to 5000 ng/ml (0.3 to 316 nM). After a 10 minute incubation with ligand, the cells were lysed and analyzed by Western blotting. The activation was determined by taking the ratio of anti-Y653/4 to anti-FGFR3 staining intensities in Western blot experiments such as the one shown in Figure 1. The wild-type activation is assigned a value of 1 at saturating *fgf1* concentration [20]. Note that this assignment is arbitrary, and the y-axis reports on the relative, and not absolute, activation.

The Western blot shown in Figure 1 was analyzed as discussed in Materials and Methods. The analysis was restricted to the 130 kDa form located predominantly in the plasma membrane, while the 120 kDa band was not included in the analysis. The activation levels for the wild-type and mutant FGFR3 were determined by calculating the ratio of the phosphostaining to total FGFR3 staining for the 130 kDa bands. The calculated activation levels as a function of ligand concentration are shown in Figure 3 with open squares for the mutant. The data (averages and standard errors) are from three independent experiments such as the one shown in Figure 1. In Figure 3, along with the data for the mutant, we also show the averaged data for the wild-type [20]. The activation level of the wild-type at saturating *fgf1* concentration is assigned a value of 1, and all measured activation levels are scaled accordingly. Note that this assignment is arbitrary, as we do not have an absolute measure of FGFR3 activation, and the y-axis in Figure 3 is “relative activation”. We see that the plateau for the mutant is 1.7±0.16 times higher than the plateau for the wild-type. Thus, the mutation increases FGFR3 activity at high *fgf1* concentrations, when all receptors are expected to be in their liganded dimeric state.

**Figure 4:**
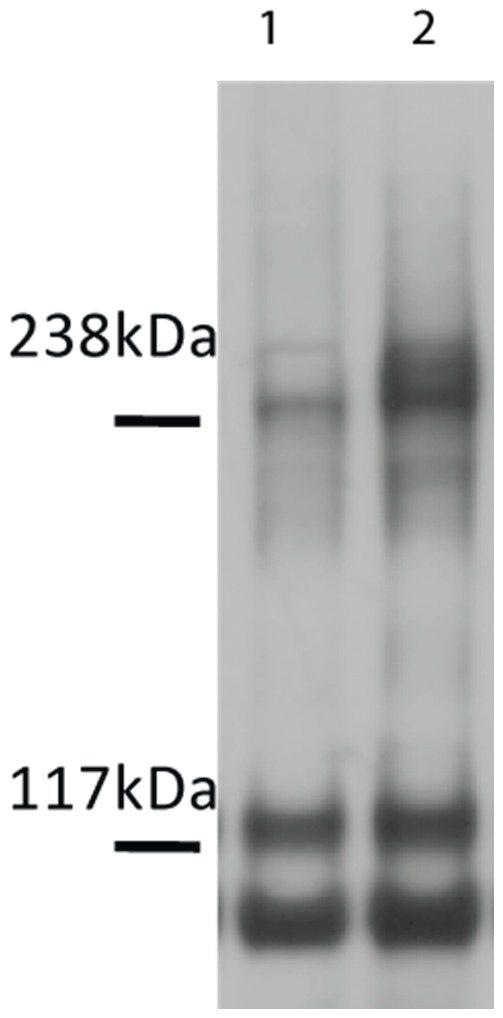
Western blots of crosslinked FGFR3/A391E in the absence of ligand and in the presence of saturating ligand concentrations. HEK293 T cells were transfected with 1 µg DNA encoding FGFR3/WT or FGFR3/A391E. Cells were cultured for 24 hours and starved for another 24 hours. Cells were incubated with BS^3^ cross-linking agent (Pierce) for 30 min at room temperature prior to lysis. The blot was stained with anti-FGFR3 antibodies (H-100, sc-9007, Santa Cruz). The crosslinked fractions were calculated by dividing the intensity of the dimer band by the sum of intensities of the dimer and the 130 kDa monomer band. **Lane 1:** no ligand; **Lane 2:** 2500 ng/ml *fgf1.* The cross-linked fraction increased from 0.35 to 0.6 upon *fgf1* addition in this experiment. Results from multiple experiments were summarized in Table 1.

A question arises if all receptors are indeed 100% dimeric in the plateau region at high ligand concentrations. One way to address this question is to use chemical cross-linking since dimeric receptors can be cross-linked [25]–[28]. However, the cross-linking reaction, as all chemical reactions, has efficiency lower than 100%, precluding us from answering this question directly. Nevertheless, here we performed crosslinking experiments with the A391E mutant using BS^3^, a membrane-impermeable crosslinker that crosslinks only the receptors in the plasma membrane in very close proximity (∼12 Å). In these experiments, HEK 293 T cells were transfected with plasmids encoding FGFR/A391E, further cultured for 24 hours, starved for 24 hours, treated with *fgf1*, and then crosslinked by BS^3^. FGFR3 was detected on Western blots using anti-FGFR3 antibodies. The results are shown in Figure 4. In the absence of ligand, we see a weak band at twice the molecular weight of mature FGFR3 (∼260 kDa) corresponding to the cross-linked mutant. The intensity of the cross-linked band increases in the presence of ligand. We quantified the bands corresponding to monomeric and cross-linked mutant FGFR3, and determined the fraction of cross-linked mutant as S_D_/S = S_D_/(S_M_+S_D_), where S_D_ is the intensity of the dimeric band and S_M_ is the intensity of the monomeric 130 kDa band. For the experiment shown in Figure 4, the cross-linked fraction increased from 0.35 to 0.6 in the presence of the ligand. The results from at least three independent experiments are summarized in Table 1, and are compared to results for wild-type FGFR3 published previously [20].

**Table 1:** FGFR3 cross-linked fractions in the absence of ligand and in the presence of 2500 ng/ml (158 nM) *fgf1*.

	− *fgf1*	+ *fgf1*
FGFR3/WT^a^	0.20±0.03	0.59±0.08
FGFR3/A391E	0.35±0.03	0.67±0.10

Data are averages and standard errors from 3 independent experiments, conducted under the transfection conditions used to measure activation. Dimeric receptors were cross-linked with the membrane impermeable crosslinker BS^3^. The increase in cross-linking due to the mutation in the absence of *fgf1* is about 75% and is statistically significant (p = 0.014). In unpublished work, we see the same increase in dimerization using FRET. The mutation does not have a significant effect on cross-linking at high *fgf1* concentrations (p = 0.21). ^a^Data from [20].

The crosslinking is very similar for the wild-type and the mutant at high *fgf1* concentration, 0.59±0.08 and 0.67±0.10 (Table 1). These data are consistent with 100% dimeric fraction and about 60% cross-linking efficiency. The difference is not statistically significant; the p-value calculated from Student t-test is p = 0.21. Since cross-linking efficiencies are expected to correlate with dimerization, the result suggests that dimeric fractions are very similar for the wild-type and the mutant at high *fgf1* concentration, consistent with the expectation that all receptors are dimeric once the plateau is reached. Yet, the activation of the wild-type and the mutant are different (Figure 3). According to equation (8), the probabilities for receptor phosphorylation within liganded dimers, Φ_D_, scale with the activation levels at high ligand concentration and are therefore also different. The ratio 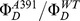 is calculated as 1.7±0.2, indicating that the mutation increases the receptor phosphorylation efficiencies within liganded dimers, Φ_D_, by 70%.

### Effect of the A391E Mutation in the Absence of Ligand

**Figure 5:**
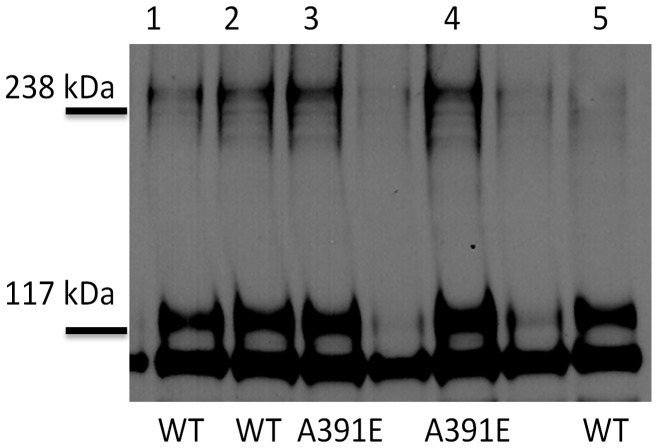
Cross-linking results for FGFR3/WT and FGFR3/A391, under conditions which allow visual comparison between the wild-type and the mutant. 2.5 µg and 4 µg of wild-type and mutant DNA were used for transfection in these experiments, in order to achieve similar expression. The lysate loading was the same in the numbered lanes, which were used for quantification. The mutation induces a modest, but statistically significant increase in cross-linked fraction from 0.28±0.03 to 0.37±0.05 (p = 0.03). The cross-linking results under the transfection conditions used in the activation studies are shown in Table 1.

It has been previously demonstrated that the mutation increases (1) the dimerization of the isolated TM domain [12] and (2) the activation of the mutant in the absence of ligand [13]. However, an increase in dimerization has not been demonstrated for the full length receptor in the plasma membrane of cells. While there are no methods to probe dimerization directly in cellular membranes, chemical cross-linking can be used to compare dimerization propensities of the wild-type and the mutant, with the caveat that small structural changes may impact the cross-linking propensities because chemical cross-linkers are reactive only to amine groups in close proximity. Here we therefore performed cross-linking experiments for FGFR3/WT and FGFR3/A391, under conditions which allow visual comparison of the results. 2.5 µg and 4 µg of wild-type and mutant DNA, respectively, were used for transfection in these experiments, yielding similar expression levels. One Western blot film is shown in Figure 5. The cross-linked fraction for the wild-type was 0.28±0.03, and the cross-linked fraction for the mutant was 0.37±0.05. The measured increase in cross-linked fraction is statistically significant: p = 0.03.

As the cross-linked fraction is expected to depend on the receptor concentration in accordance with the law of mass action (equation 1), we next performed cross-linking experiments under the transfection conditions of Figures 1 and 3, using 1 µg of DNA for transfection. From 3 independent experiments, we determined the cross-linked fractions as 0.20±0.03 for the wild-type and 0.35±0.03 for the mutant (Table 1). This is an increase by 75% due to the mutation, in the absence of ligand. In unpublished work, we see the same increase in dimerization using FRET (not shown).

At zero ligand concentration, in Figure 2 we see that the activation of the mutant is about 3 times as high as the activation of the wild-type. Thus, both cross-linking and phosphorylation are increased due to the mutation. However, the activation increase (∼ 3 times, Figure 3) is larger than the increase in cross-linking (∼ 1.75 times, Table 1). Assuming that cross-linking correlates with dimerization, it can be concluded that the increase in dimerization propensity is not the sole reason for the activation increase. The mutation also increases Φ_d_, the probability for receptor phosphorylation within the unliganded dimers. With the help of equation (11), written for both the wild-type and the mutant, the ratio 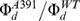 was calculated as 1.69±0.20. Thus, Φ_d_ increases by about 70% due to the mutation, similarly to Φ_D_ (see above). Therefore, the mutant dimeric receptors are easier to phosphorylate than the wild-type, both in the absence and in the presence of ligand.

### Calculation of K_1_ and K_2_ for Mutant FGFR3

Additional insight about the effect of the A391E mutation on FGFR3 activation can be gained by fitting the physical-chemical model described by equations (3) through (12) to the experimental data. To perform this analysis, we first calculated the ratio Φ_d_/Φ_D_ for the mutant using equation (12). Since the phosphorylated fraction at zero ligand concentration 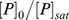 is 0.35±0.04, the ratio (Φ_d_/Φ_D_) for the mutant can be calculated as 0.67±0.10. Next, we estimated the average expression of the mutant in the experiments by comparing it to FGFR3 expression in the stable cell line HEK293-fWT expressing about 8.4±1.3 x 10^5^ receptors per cell [17]. A comparison using Western blots yielded the average concentration, [*TR*], of the mutant as 7.0 × 10^4^ copies/cell (for comparison, [*TR*] = 1.76 × 10^5^ for the wild-type).

**Figure 6:**
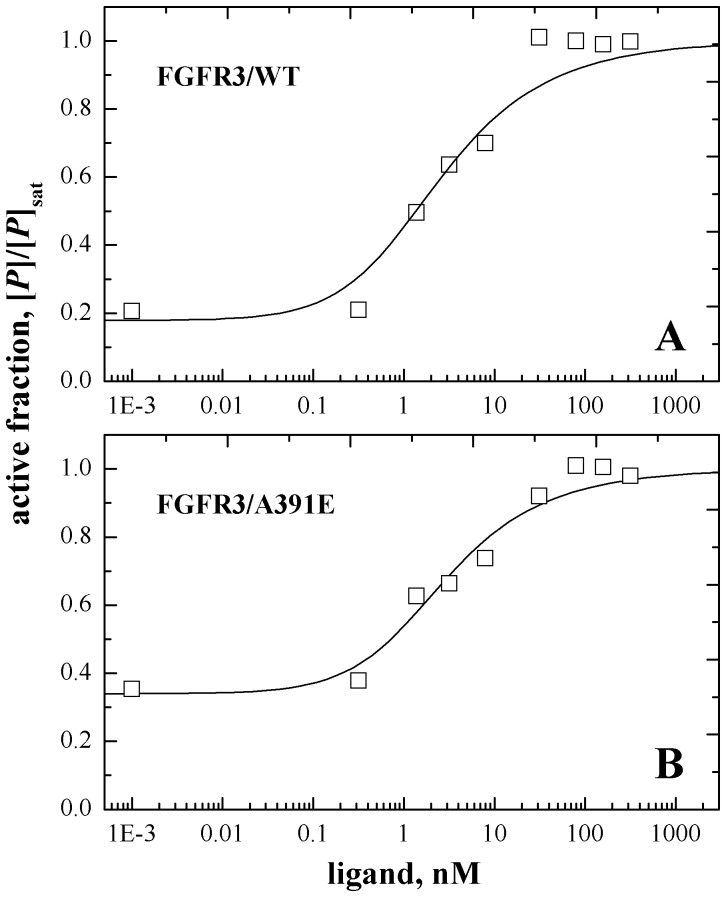
Fits of the RTK activation model, given by **equations (3**) through (9), to the Western blot data. Each data set is from three independent experiments. Data from Figure 3 are repotted, such that the activation plateau is assigned a value of 1. This renormalization yields the data as “active fractions” which can be then fitted to the theoretical prediction given by equation (7). The two reaction constants, *K_1_* and *K_2_*, were varied to achieve the best fit to the data. Fit results are shown in Table 2. (A) Results for wild-type FGFR3 [20]. (B) Results for the A391E mutant.

To fit the experimental data using the above values for [*TR*] and (Φ_d_/Φ_D_) as fixed parameters, we first re-plotted the mutant activation data, such that the activation at saturating *fgf1* concentrations is set to 1. As discussed previously, this re-normalization yields the data as “activated fractions” [13], [17], [20]. We then fitted the prediction of the activation model given by equations (3) through (12) to the measured activated fractions using a Matlab™ code. There were two unknowns in the fit, *K_1_* and *K_2_*. The constant *K_1_* reports on the dimerization propensity of the receptor and the constant *K_2_* is a measure of the strength of ligand binding to the receptor. Initial guesses for these unknown parameters were inputted into the Matlab™ code, which calculated [*d*], [*D*] and [*M*], and the prediction for activated fractions. This prediction was compared to the experimentally determined activated fractions and the two unknowns *K_1_* and *K_2_* were varied until the calculated predictions of 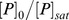 provided the best description of the experimental data. The fit for the mutant is shown in Figure 6B, and the results for the wild-type [20] are shown in Figure 6A for comparison. The optimal parameters determined in the fits are shown in Table 2. The results do not depend on the initial conditions, indicative of robust fits. We see that the ligand binding constants for the wild type and the mutant are similar. Thus, the mutation does not affect ligand binding. On the other hand, the A391E mutation induced a change in the apparent dimerization free energy: ΔΔG = −1.3±0.4 kcal/mol.

**Table 2:** Optimal values for the apparent dimerization free energy (ΔG = -RTlnK_1_) and the apparent ligand-dissociation constants (1/*K_2_*), determined by fitting the theoretical predictions of active fractions, given by equations (3) through (12), to the experimentally measured active FGFR3 fractions (fits shown in Table 5).

	ΔG = -RTlnK_1_ (kCal/mol)	1/K_2_ (nM)
FGFR3/WT^a^	−3.70±0.40	0.2±0.1
FGFR3/A391E	−4.95±0.20	0.3±0.1

a Data from [20].

## Discussion

Many RTK pathogenic mutations have been reported to enhance RTK activation, and the degree of activation has been shown to correlate with the severity of the phenotypes [8], [9], [27], [29]–[31]. Consistent with this view, FGFR3 carrying the A391E mutation exhibits higher activation than wild-type FGFR3. In particular, here we show that the A391E mutation increases FGFR3 activation in the absence of ligand, as well as in the presence of ligand (Figure 3). Thus, it can be expected that the pathology in Crouzon syndrome with Acanthosis Nigricans is directly linked to the elevated activation of mutant FGFR3.

An increase in RTK activation in the presence of a single amino acid mutation could arise due to different factors, such as increased dimerization, increased ligand binding, or alterations in the structure and activity of the kinase domain [32]–[35]. To gain insight into the cause for the enhanced activation due to the A391E mutation, here we studied the effect of the mutation on FGFR3 activation and on FGFR3 cross-linking, and we analyzed the data using a physical-chemical model of FGFR3 activation. Our results are summarized and discussed below.

### Effect of the A391E Mutation on FGFR3 Dimerization

Many TM domain mutations in FGFR3 are believed to increase FGFR3 activation due to increased dimerization. Examples include the G370C, S371C and Y373C mutations implicated in thanatophoric dysplasia type 1 (TD1), expected to stabilize the FGFR3 dimers via disulfide bonds [36]. Here we find that the A391E mutation enhances the crosslinking propensity of FGFR3 in the absence of ligand (Figure 5). Furthermore, by fitting the model given by equations (3) through (12) to the experimental data, we demonstrate that the mutation increases FGFR3 dimerization propensity by −1.3±0.4 kcal/mol.

The idea that the Glu391 mutation stabilizes the FGFR3 dimer has been proposed previously. Originally, the speculation was based on results for isolated FGFR3 TM domain peptides incorporated in lipid vesicles [12]. Later, chimeric Neu receptors containing the wild-type and mutant FGFR3 TM domains were investigated [18]. These studies suggested that the mutation stabilizes the FGFR3 dimer, but none of these studies were conducted with full-length FGFR3. Recently, the propensity of full-length FGFR3 for ligand-independent activation was measured, and the effect of the mutation of ligand-independent activation was calculated as ΔΔG = −1.7 kcal/mole [13]. This value was similar to the previous FRET measurements of interactions between the isolated transmembrane domains in liposomes ΔΔG = −1.3 kcal/mole, and thus it was proposed that the increase in ligand-independent activation is solely due to dimerization [13]. Here we show, however, that the increase in dimerization does not account completely for the increase in ligand-independent activation, as the probability for phosphorylation at the critical tyrosines in the activation loop is also increased due to the mutation.

### Effect of the A391E Mutation on Ligand Binding

Mutations in FGFR3 have been shown to cause pathologies by affecting ligand binding [37]. For example, the P250R mutation in FGFR3, causing Muenke syndrome, increases ligand binding [38], [39]. While most of the mutations that affect ligand binding occur in the extracellular domains, the G380R mutation in FGFR3 TM domain has also been proposed to alter ligand binding [40]. Here we find that the strength of *fgf1* binding to FGFR3 is not affected by the A391E mutation, suggesting that the mutation does not affect the conformation of the extracellular domain. However, at this point, the possibility that the mutation affects receptor binding to other *fgf* ligands cannot be ruled out.

### Effect of the A391E Mutation on FGFR3 Dimer Phosphorylation Levels

Some RTK mutations lead to pathologies by causing structural changes that increase the activity of the kinase domains [41], [42]. In this case, the mutant dimers are more active than the wild type dimers, and RTK over-activation occurs despite the fact that the number of dimers is not increased. This mechanism of over-activation was recently demonstrated for the G380R mutation in FGFR3 linked to achondroplasia [17], [21]. The data and the data analysis presented here suggest that the efficiencies of phosphorylation at Y653/654 in both unliganded and liganded FGFR3 dimers (i.e. Φ_d_ and Φ_D_) increase due to the A391E mutation. Thus, besides increasing dimerization, the A391E mutation most likely induces a structural change that facilitates the cross-phosphorylation of these tyrosines.

Here we emphasize that the efficiencies of phosphorylation at Y653/654 in both unliganded and liganded FGFR3 dimers (i.e. Φ_d_ and Φ_D_ in equation (5)) are quantities that are not measured directly in experiments. The measured activation in Figures 1 and 3 depends both on the dimerization propensities and on the phosphorylation probabilities Φ_d_ and Φ_D_ within dimers. The physical-chemical approach used here to analyze the data allows us to decouple the dimerization propensities and the phosphorylation probabilities, and thus delineate the effect of the mutation on different steps in RTK activation.A published molecular model of the isolated FGFR3 TM domain dimer, created with the program CHI, predicts that the mutation does not alter substantially the structure of the TM dimer [12]. However, Glu391 is at the C-terminal flank of the TM domain, and the hydrogen bond between Glu391 and the other receptor in the dimer may affect the link between the TM domain and the catalytic domain. It has been shown that there is a “rotational coupling” between the TM domains and the catalytic domains in RTKs, such that changes in the C-terminal flank of the TM domain dimer may impact the orientation of the kinase domains with respect to each other [43]. Thus, while the exact structural details are unknown, it is possible that the Glu391-mediated hydrogen bonds affect the activity of FGFR3 kinase domains.

### Effect of the A391E Mutation on FGFR3 Expression

Pathogenic FGFR3 mutations are known to impair FGFR3 trafficking and downregulations [8], [26], [44], [45]. The expression of the mutant is lower than the expression of the wild-type in HEK 293T cells under identical transfection conditions, as shown here and in reference [13]. While the exact mechanism behind this decrease in surface expression is not clear, this effect leads to a decrease in the number of active mutant receptors on the cell surface. This mechanism works to attenuate signaling, counteracting the mutation-induced increase in the number of dimeric receptors (due to increased *K_1_*) and the increase in dimer activity (due to increased Φ_d_ and Φ_D_). The signal attenuation due to decreased surface expression may explain the relatively mild phenotype in Crouzon syndrome with Acanthosis Nigricans.

### Conclusion

Here we use a physical-chemical approach that allows us to decouple the dimerization propensities and the phosphorylation probabilities of RTKs, and thus delineate the effect of the A391E mutation on different steps in FGFR3 activation. The A391E mutation has been previously suggested to cause pathology due to increased FGFR3 dimerization. We confirm this effect, but we also demonstrate that the increase in dimerization does not account completely for the increase in activation. The mutation also facilitates the phosphorylation of critical tyrosines in the activation loop, via a mechanism that is currently unknown. Thus, pathogenesis is likely linked to both (i) increased dimerization and (ii) increased phosphorylation levels of both unliganded and liganded dimers. While these two different effects work synergistically to increase FGFR3 activation, the A391E mutation also decreases the surface expression of the receptor, attenuating signaling. The latter effect may be a determinant of the mild phenotype in Crouzon syndrome with Acanthosis Nigricans. The results presented here emphasize the complexity in FGFR signaling and provide new insights into how pathogenic mutations cause human diseases.

## Supporting Information

Methods S1 (DOCX)

Figure S1 **Western Blot band intensities as a function of FGFR3 loading. (A): anti-FGFR3 antibodies. (B): anti-P-Y653/4 antibodies.**
(TIF)
