# Supplementary material for: Multiple Consequences of a Single Amino Acid Pathogenic RTK Mutation: The A391E Mutation in FGFR3
Source: PLoS One. 2013 Feb 20;8(2):e56521. doi: 10.1371/journal.pone.0056521 (PMC3577887; doi:10.1371/journal.pone.0056521)
Supplement: Methods S1 — (DOCX) [file pone.0056521.s001.docx]

**SUPPLEMENTAL INFORMATION**

**Supplementary Methods S1**

Western blot artifacts can arise if the intensity of the band is too low (such that no bands can be observed and quantified), or too high (such that saturation occurs). We therefore determined the low and high limits of band intensities that bracket the so-called “linear staining regime” (1-3). HEK 293T cells were transfected with 1 μg DNA of FGFR3 in the pcDNA vector. 24 hours after transfection, the cells were starved for 24 hours and then treated with 100 ng/ml fgf1. The cells were lysed after 10 minutes and a large stock of lysate was prepared referred to as the “grand control”. Western blots of the “grand control” were run as described in Materials and Methods, while varying the amount of lysate loaded per lane. After the proteins were transferred onto a nitrocellulose membrane, the bands were stained using anti-FGFR3 antibodies (for detection of all receptors) or anti-Y653/4 antibodies (for detection of receptors that are phosphorylated). The results of these experiments are shown in Figures S1A (for anti-FGFR3 antibodies) and S1B (for anti-P-Y653/4 antibodies).

Figure S1A shows the intensity of the bands, stained using the anti-FGFR3 antibody which detects all FGFR3 receptors (both active and inactive), as a function of amount of lysate loaded. The results are linear if the anti-FGFR3 band intensity is lower than 1.3 x 10^6^. Similarly, Figure S2B shows the intensity of the bands when the anti-P-Y653/4 antibody was used. We see that the results are linear up to anti-P-Y653/4 band intensities of 1.7 x 10^6^. Based on the results shown in Figure S1, we adjusted the loading in all experiments, such that the anti-FGFR3 band intensity did not exceed 1.3 x 10^6^ (and was above 2.5 x 10^5^), and the anti-P-Y653/4 antibody staining intensity did not exceed 1.7 x 10^6^ (and was above 2.5 x 10^5^), while following the protocol used in the calibration experiments. Most importantly, on each gel we also ran 2.5 μl of the “grand control” lysate stock, to ensure that its band intensity is the same as in the calibration experiments. This procedure ensured that all bands are within the “linear regime”.
