# Supplementary figures and images for: Multiple Consequences of a Single Amino Acid Pathogenic RTK Mutation: The A391E Mutation in FGFR3
Source: PLoS One. 2013 Feb 20;8(2):e56521. doi: 10.1371/journal.pone.0056521 (PMC3577887; doi:10.1371/journal.pone.0056521)

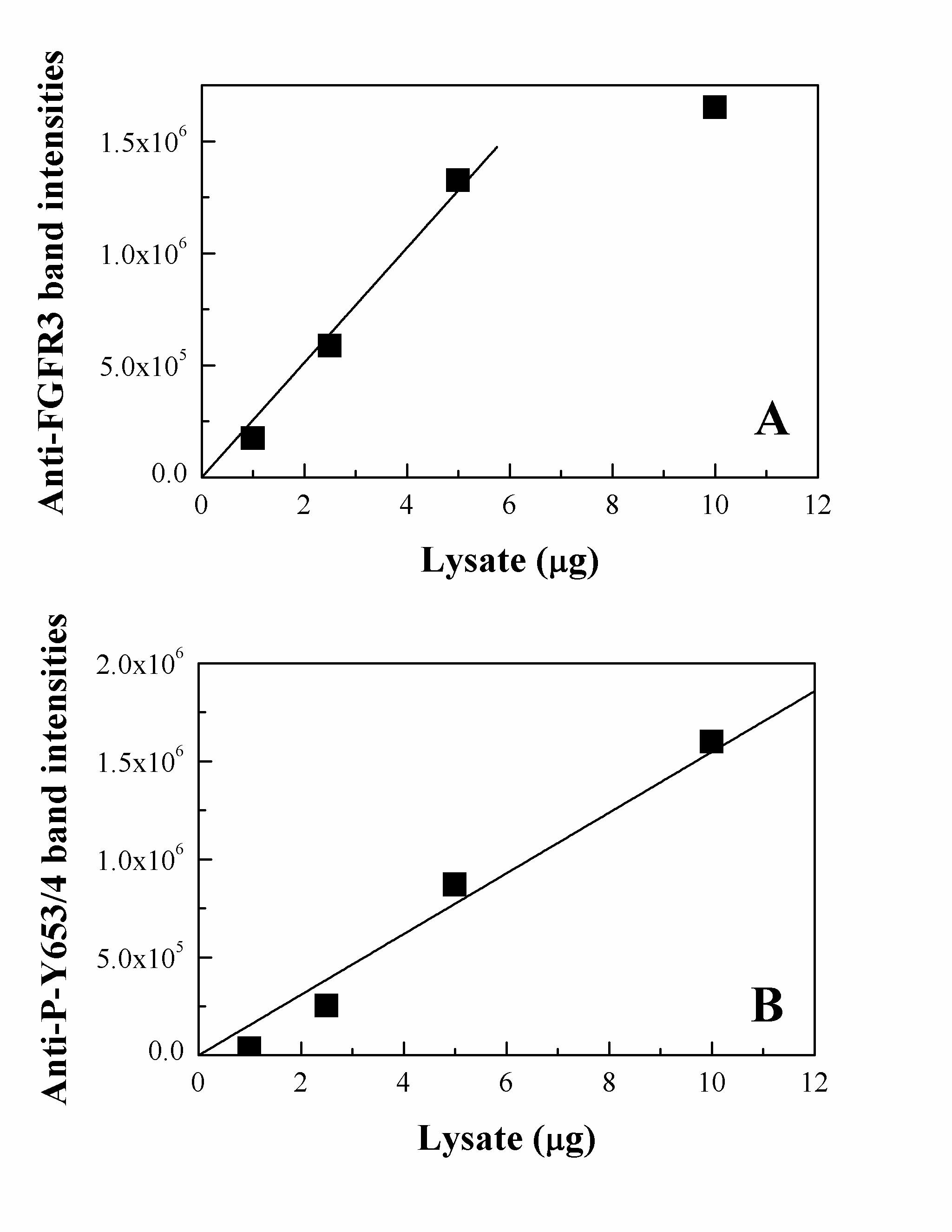

Supplement: Figure S1 — Western Blot band intensities as a function of FGFR3 loading. (A): anti-FGFR3 antibodies. (B): anti-P-Y653/4 antibodies. (TIF) [file pone.0056521.s002.tif]
